# “The thing is, kids don’t grow the same”: Parent perspectives on preschoolers’ weight and size in Soweto, South Africa

**DOI:** 10.1371/journal.pone.0231094

**Published:** 2020-04-06

**Authors:** Sonja Klingberg, Esther M. F. van Sluijs, Catherine E. Draper

**Affiliations:** 1 MRC Epidemiology Unit, UKCRC Centre for Diet and Activity Research (CEDAR), University of Cambridge, Cambridge, United Kingdom; 2 SAMRC Developmental Pathways for Health Research Unit (DPHRU), School of Clinical Medicine, University of the Witwatersrand, Johannesburg, South Africa; Newcastle University, UNITED KINGDOM

## Abstract

The prevalence of overweight and obesity is high among preschool age (3–5 years) children in South Africa, and children in urban low-income settings are particularly at risk. A better understanding of how parents or caregivers of young children perceive children’s weight and size, as well as contextual factors influencing perceptions, is needed to inform interventions. The aim of this study was to examine how parents of preschool children in Soweto, South Africa, view childhood obesity, and to situate these perspectives in the context of the home environment in which preschool age children in Soweto live. Semi-structured in-depth interviews were conducted with 16 parents in four neighbourhoods of Soweto. Interviews were audio-recorded, transcribed verbatim, and analysed using reflexive thematic analysis following a contextualist approach. Three themes were developed: growing differently, the ‘right’ way to be, and weight is not health. These themes capture parents’ views on complex and reportedly inevitable causes of obesity, ideas about acceptable and preferred body sizes, and the low priority of weight *per se* compared to health. The findings suggest that childhood obesity prevention in South Africa needs to be done in a non-stigmatising way that recognises environmental and contextual factors, such as parents’ limited sense of agency in relation to their children’s health and weight, and concrete resource constraints. Environmental barriers to healthy behaviours need to be addressed in order to overcome the coexisting challenges of childhood undernutrition and obesity in urban low-income South African settings.

## Introduction

Childhood obesity is a relatively recent but growing public health issue in South Africa, and many other African settings [1–4]. In the Southern African region, the prevalence of obesity among under 5-year-olds is estimated to be 13%, which is more than double the mean prevalence for the entire continent [1]. In South Africa, a national survey from 2013 suggests an even higher combined prevalence of overweight and obesity in 2-5-year-old children at 22.9% [5]. Obesity in early childhood is concerning because it is associated with health and psychosocial problems both in childhood and later in life, and it is likely to track into adolescence and adulthood [6–11]. Children living in urban low-income settings in South Africa have been identified as being particularly at risk when it comes to childhood obesity [12–14].

Much of the literature on childhood obesity prevention in African settings focuses on older school-age children [15], and there is a need to better understand the circumstances under which obesity develops in the earlier years of childhood. Moreover, perceptions and behaviours related to childhood obesity in low- and middle-income countries have not received much attention in existing qualitative literature, particularly in younger age groups. For example, a review of qualitative studies examining barriers and facilitators to preschool age children’s physical activity found such qualitative research from low- and middle-income country contexts largely missing [16].

It is important to note that in Southern Africa, overweight and obesity coexist with food insecurity and persistent rates of undernutrition in childhood, with the prevalence of stunting in the under-5 age group at 29.3% [1]. Despite South Africa being an upper-middle-income country, many families experience similar levels of poverty or deprivation as countries classed as low-income as South Africa has one of the highest levels of income inequality in the world [17]. This income inequality still largely follows the racial patterns deliberately engineered through apartheid policies between 1948 and 1994.

Qualitative research involving South African adolescents has shown that there are complex, and sometimes conflicting, social and cultural norms and ideals related to body size and behaviours such as physical activity and healthy eating [18–22]. Previous research in South Africa has also explored views of child-minders and health workers when it comes to childhood obesity in younger children [23]. However, little is known about parental views on childhood obesity and preschool age children’s health behaviours in South Africa. Given that parents and caregivers are relied upon to recognise issues and take action when it comes to children’s health and well-being, it is important to elicit their views on children’s weight and size in specific contexts. Such qualitative work in Indonesia has found, for example, that carers believe”chubbier” children to be healthier [24], and a study from South Africa also identified perceptions of obesity being associated with wealth and power [23]. However, in the absence of more comprehensive research on such preferences, it is unclear whether this is reflected in the views or practices of South African caregivers of young children.

The aim of this study is to describe how parents or caregivers of young children in Soweto, South Africa, view childhood obesity, and to situate these perspectives in the context of the home environment in which preschool age children live. The study design is qualitative, drawing on the experiences and perspectives of parents with a child or children of preschool age (3–5 years) in Soweto.

## Methods

### Theoretical approach and study design

The rationale for conducting in-depth qualitative research on caregiver perspectives and home settings of preschool age children is to inform future interventions targeting young children in urban low-resource settings in South Africa. Since obesity is a complex public health issue, qualitative research provides opportunities to gain deep, context-specific insights about the individual, family, social, and environmental factors contributing to obesity. This study is therefore situated within a contextualist epistemological approach to qualitative inquiry, and utilises semi-structured in-depth interviews, field observations and reflexive thematic analysis [25–27]. This approach was selected to enable a focus on individual experiences and perceptions in a way that recognises how the broader social and physical environment may constrain, enable, shape or be shaped by, the individuals, zoomed in on through the qualitative inquiry. As described by Braun and Clarke [27], a contextualist application of thematic analysis is influenced by critical realist theory that “acknowledge[s] the ways individuals make meaning of their experience, and, in turn, the ways the broader social context impinges on those meanings, while retaining focus on the material and other limits of ‘reality’.”(p.81) Essentially, what people say is taken to be a reflection of the reality that they are living and experiencing, and where possible, individual accounts are contextualised by providing a rich description of the environment in which study participants live, and any relevant interactions between the individual and other levels of the social-ecological model of health [28], such as family circumstances or community characteristics.

### The study setting

Soweto is a densely populated urban township that forms part of the City of Johannesburg Metropolitan Municipality in Gauteng Province, South Africa. Colonial and apartheid urban planning policies of racial segregation assigned most of Soweto to Black African residents [29], and the population of Soweto is still predominantly of Black African descent. Unemployment is high, and there are numerous squatter communities and informal settlements, consisting of shacks or other informal housing with very limited basic amenities. However, there are also areas that have become more middle-class.

The neighbourhoods included in this study represent relatively formal and socioeconomically diverse parts of Soweto, with most participants living together with extended family in typical detached houses with electricity and running water that also often have additional rooms or shacks for family members or tenants in the backyard. Some participants were tenants in such rooms or shacks, and typically employed and less dependent on extended family as the other participants living in family houses. The socioeconomic circumstances of study participants varied somewhat, ranging from participants who were unemployed and without much family support or state welfare, to participants who were employed or studying while living with family members who had a stable income.

The most noticeable differences between the neighbourhoods were related to traffic and resulting safety concerns. Traffic-related safety concerns were prominent because of the high rates of injury and death in South Africa resulting from road traffic accidents, often due to drunk or otherwise reckless driving [30]. All homes had some kinds of fences, yards, and gates. Some participants lived on quieter or more spacious streets where children frequently played outside the gates, while others had bigger yards and less need for space outside the gates. Some, however, had very little space for children to play outside the house at all. The neighbourhoods were not noticeably different in terms of other safety concerns beyond traffic.

### Participants and recruitment

In South Africa, preschools are not part of the compulsory and free basic education system, and early childhood development programmes before the age of five are provided by private day care centres and preschools. This means that parents pay fees for their children to attend preschool, and as such not all children access pre-primary education. Currently, 69% of South African children in the 3–5 years age group attend some form of early learning group programme [31]. Nevertheless, recruitment through preschools was undertaken as these could facilitate contact with families that had children in the age group of interest. Six preschools were approached with information about the study, and principals from four of them agreed to passing on information and facilitating contact with caregivers of 3-5-year-old children. The other preschool principals were reportedly too busy to get involved in the research, or they did not respond. Sixteen primary caregivers of preschool age children were purposively recruited via the four different preschools in four neighbourhoods in Soweto, South Africa. Characteristics of the study participants are described in Table 1. New participants were recruited gradually until there was sufficiently rich and diverse data to answer the study’s research questions. Out of the 16 participants, 14 were biological mothers of at least one preschool age child. Two participants were biological fathers of a preschool age child. A conscious effort to recruit fathers into the study was made but the vast majority of both potential and actual participants were women. All participants were of Black African descent but represented different ethnic and linguistic groups of South Africa. Their preschool age children attended preschool Monday to Friday, from 8 o’clock in the morning to somewhat different hours in the afternoon or evening depending on the parents’ work or other commitments.

**Table 1:** Sociodemographic characteristics of study participants.

#	Age	Relationship to preschool child(ren)	Marital status	Age of preschool child(ren)	Highest level of education	Employment status	Social grants in household	Neighbour-hood
**1**	21	Mother	Single	4	CSE	Unemployed	Child support	1
**2**	26	Mother	Single	4	CSE	Employed	Child support	2
**3**	30	Mother	Married	4	CSE	Unemployed	None	2
**4**	38	Mother	Divorced	4	CSE	Employed (part-time)	Child support	1
**5**	47	Mother	Widowed	5	CSE	Employed	None	1
**6**	23	Mother	Single	4	SSE	Unemployed	Child support	1
**7**	30	Mother	Married	4	CSE	Employed	None	1
**8**	42	Mother	Single	3 & 4	SSE	Unemployed	Child support	3
**9**	36	Mother	Single	5	CSE	Employed	Child support, relative’s disability grant	1
**10**	37	Mother	Single	4	CSE	Employed	Child support	3
**11**	27	Mother & aunt	Single	4 (son) & 3 (niece)	CSE	Unemployed	Unclear	1
**12**	25	Mother	Single	4	TE	Recently unemployed (occasional work)	Child support	4
**13**	25	Mother	Single	4	CSE	Student	None	1
**14**	24	Father	Single	4	TE	Student (part-time work)	None	1
**15**	29	Mother	Married	5	CSE	Employed	Older child’s disability grant	1
**16**	37	Father	Single	5	TE	Unemployed	None	1

CSE: Completed secondary education; SSE: Some secondary education; TE: Tertiary diploma or certificate. Neighbourhood 1: Generally quiet, small streets and cul-de-sacs with some space to play in the street; Neighbourhood 2: Very busy roads, small yards and no space to play outside the gates; Neighbourhood 3: Busier streets than neighbourhood 1 but space to play inside yards, and outside the gates in some cases; Neighbourhood 4: Varying traffic depending on location, generally no space outside yards. Some children from neighbourhood 1 attend preschool here.

After providing information about the study, and opportunities to ask questions, written consent was recorded from each participant wishing to participate, and separate written consent was obtained for audio recording the interviews. Each participant was also given a R150 (approximately US$10) supermarket voucher at the end of the interview as appreciation of their time, but this was not mentioned in the information sheet or verbally before the interview had been completed so as not to incentivise or pressure people into participating. Some interviews were rescheduled, even several times, and some were cancelled due to potential participants’ other time commitments. Not having time to participate was the only reason given for refusals.

### Data collection

In-depth interviews were conducted by the first author in English in the participants’ homes, or in a location convenient for them, such as a relative’s home, between September 2018 and January 2019. A local Sowetan fieldworker with good proficiency in several of the official languages spoken in South Africa in addition to English facilitated contact with preschools and participants, and was present for each interview to assist with data collection. The fieldworker had received basic training in qualitative research methods and ethics.

A pilot-tested topic guide was utilised flexibly in the interviews (S1 File). New questions and topics were added to the guide as they emerged throughout the interviews. A questionnaire was also used to collect sociodemographic and other background information (S2 File). Some items, such as an observation checklist for toys and sports equipment found in the home, were dropped as they were not conducive to a relaxed, friendly and respectful interview atmosphere. In addition to the audio recorded interviews and the background questionnaires, contextualising field notes were compiled by the first author based on observations about the interview visit, the participant’s home and neighbourhood, and the interactions between the participant and the preschool age child if they were present during the interview. Field observations were also discussed together with the fieldworker.

The interview recordings ranged from 28 to 83 minutes in length. Later interviews were longer due to new questions and topics arising and being added to the topic guide. Interview recordings were transcribed verbatim by a professional transcriber (n = 12) and checked by the first author, or transcribed verbatim by the first author (n = 4).

### Data analysis

Analysis of transcripts was carried out using reflexive thematic analysis according to the process developed by Braun and Clarke [25–27], and the fieldnotes were used to support and contextualise the analysis. This qualitative analysis method was selected because its reflexive nature and compatibility with a contextualist approach enabled analysing individual perspectives in an open, inductive and contextually grounded way.

This particular method of thematic analysis consists of six phases, which do not necessarily occur in a linear or discrete manner. The first phase of familiarising oneself with the data comprised transcription, checking transcripts, and repeated reading of transcripts. The second phase involved generating initial codes, and organising these into categories (domains) according to whether they related to childhood obesity, specific behaviours, parenting or other topics. This categorisation was a practical rather than analytical exercise, facilitating the analysis through making the codes easier to navigate. In the third phase, preliminary themes were developed and discussed. These were reviewed, reorganised, defined and named in the fourth and fifth phases. Finally, the sixth phase comprised summarising and describing the themes.

An inductive approach to analysing the manifest content of interview data was taken in order to be open to new concepts or patterns arising throughout the interviews, and to avoid potentially misguided latent interpretation in this cross-cultural qualitative inquiry.

### Ethical approval

Ethical approval was obtained from the University of the Witwatersrand’s Medical Human Research Ethics Committee (reference number: M180257), and this approval was also supported by the Psychological Research Ethics Committee at the University of Cambridge in the United Kingdom.

## Results

Through the thematic analysis process described above, three themes that capture meaningful patterns of relevance to the aim of this study were developed. They relate to different aspects of children’s growth, size, behaviours and family situations.

### Growing differently

This theme captures how parents in the study talked about differences in children’s body weights and sizes. They had different ideas about how children grow, and why some children are bigger or smaller than others. Their views around children growing differently, and whether they were more concerned about underweight or overweight tended to be connected to how they perceived their own preschooler to be growing. Overweight was rarely a concern people had regarding their own children, even if some considered it to be very common among children in their neighbourhood.

*“The thing is um*, *kids don’t grow the same*. *You might feed them whatever you feed them the same amount of food and whatever but from their birth ah they’re not the same cause you get a child which weighs three point what*, *what*, *or six point what so they won’t grow the same*. *Others are going to be thin*.*”* (Interview 5, mother)

Across interviews, many different explanations were offered for why children are different in terms of height or weight. Parents cited the role of genes or family background, and that children and their bodies just are a certain way for no particular reason, as explanations to children growing differently. As several of the quotes illustrate, parents did not necessarily or consistently see a direct link between how much children were eating and how they were growing, and so other factors were seen to be involved in determining a child’s weight regardless of eating habits.

*“I don’t know if this makes sense*, *but his body*, *like the way he’s structured*, *it’s not*, *he’s not someone that would like get fat*. *I don’t know*, *even if he eats a lot*.*”* (Interview 14, father)

*“I’m thinking maybe it’s genetic also because even in his class there are kids that are… huge*. *When you look at them*, *they’re like seven*, *eight years* … *It’s just a genetic thing*.*”* (Interview 9, mother)

*“Unless that’s genetic of course*, *there are those people that*, *it just runs in the genes*. *No matter how good or badly their situation is*, *but it’s just in the genes*. *They’re just fat*.*”* (Interview 16, father)

*“Others are born like that*, *you’re overweight and having nothing to eat*, *it’s your body*, *it’s like that you know*, *and you’re underweight and you’ve got everything*. *You have a plate of food every day*, *you’ve got a cereal in the morning*, *during the day you do have snack*, *you’ve got everything but your body it’s like so thin*, *it’s your body like that*. *Others have got nothing but their body is so huge and those people you find that they even go to bed without anything*, *but it’s their body*, *it’s like that*.*”* (Interview 5, mother)

Different stages of growth were also acknowledged, and many described “baby fat” as a normal stage, likely to pass as children reached preschool age. Monitoring a child’s growth was usually done by observing how they looked compared to other children their age, and how their clothes fit. Some observed with relief that their children had suddenly grown in height, or gained a better appetite. Children being thin or short for their age worried parents, and this was often attributed to a poor appetite in one’s own children, and parental neglect or problems in the home when it came to other children. The reasons parents gave for children growing differently thus only selectively placed blame on parents or circumstances, and tended to focus on biological or otherwise unmodifiable factors.

### The ‘right’ way to be

Discussing the language used to describe children’s weight or size revealed both some personal preferences that parents had, and more general norms regarding the ‘right’ way to be. According to the parents in the study, both underweight and overweight might be commented on by strangers, other children or parents, but not necessarily in the same way. Underweight was more consistently described as worrying or attracting negative comments or concerns about parental neglect. Overweight, on the other hand, could receive positive attention. Parents tended to be either sceptical about the concept of ‘normal weight’, or unsure about what it meant. Instead, the term that was used to capture a desired size or appearance was “fresh”. What this meant in practice was not about a specific weight but being “fresh” implied not looking too thin, and having enough to eat.

*“It's not supposed to be fat*, *it's supposed to be fresh*. *There's fat*, *fat is obese*. *It is a big baby*, *a very big baby*. *And then*, *the fresh child is a child…where you can't see all the bones*. *My son*, *I can see the bones*, *he worries me [laughing]*. *Ja*.*”* (Interview 10, mother)

However, the word “fresh” also came up when discussing children’s overweight and obesity as a way to describe the way a child’s body is “supposed to be”, which was distinct from obesity or being fat in a negative sense. Being “fresh” could mean being round, big or “chubby” but not to the point of being somehow negatively affected by the weight or looking older than other children, implying that obesity, on the other hand, meant an unhealthy or very noticeable level of excess weight. “Fresh” was described as a positive word but preferences around size or the ‘right’ way to be were described as not necessarily being connected to health. This is elaborated further in the third theme.

One mother described both others and herself liking the way “chubby” children look but she did not think that should be something to aspire to, and criticised parents for letting their preferences about cuteness or positive comments from others lead to what might be an unhealthy body size.

*“Don’t take other people’s saying and make it yours*, *you know that your child is big…do something*. *She might be looking nice*, *yes*, *you’re chubby*, *nice*, *nice*, *nice…but where does that fatness comes from*? *You see*, *you must do something*.*”* (Interview 9, mother)

A child’s size or weight could be read in many ways, and thus the ‘right’ way to be was not one specific thing, but rather depended on what different parents prioritised. Underweight attracted concerns and negative attention, whereas children’s overweight or obesity could also be attributed to different circumstances in the child’s family, including affluence and a child having everything they want or need, as well as parents not knowing or caring about health risks associated with obesity. In addition to looking nice or representing the ‘right’ size for a child, being “fresh” was also associated with happiness and wealth.

Interviewer: “*Okay*, *and you mentioned fresh*, *what does that mean*?*”*

Participant: “*The child is living well*, *like they can get everything they want*, *that's how they rephrase it*.*”* (Interview 1, mother)

The ‘right’ way to be centred on not being too different from other children, at least not in a negative way, and parents expressed concerns about stigma and bullying if children were perceived to be different. Some of the most educated parents in particular also talked about children’s body image and self-esteem, and the importance of ensuring that children were taught to be confident about who they are, and not affected by other people’s comments.

*“Sometimes it helps…the child to be much more resilient and not take offences easily because it’s… out there it’s a cruel world*. *It’s a terrible world*.*” (*Interview 12, mother)

### Weight is not health

Interviews easily moved from the topic of weight or growth to other aspects of health, such as infectious childhood illnesses. This theme represents the ways in which a distinction was made between children’s weight and their health. As one of the fathers pointed out, weight is not the same as health because being fat does not necessarily mean being unhealthy or healthy.

“*Fat does not mean unhealthy*. *Fat does not mean healthy*, *as well*.” (Interview 14, father)

A child could be healthy or unhealthy regardless of their weight, and health mattered more than weight to the parents in the study. Discussions around what looking healthy meant often focused more on signs of illness in children’s behaviour, eyes or skin than observing their weight or growth. What people described as unhealthy foods were not necessarily problematised because of how they might affect children’s weight, but rather because of poor food hygiene, causing hyperactivity, negative effects on appetite, and health consequences like diabetes and hypertension.

*“It depends on what they eat mostly*, *um yeah mostly what they eat that makes them healthy*, *cause mostly if you keep on feeding a child junk food it’s gonna be a problem*. *It’s gonna give them maybe high hypertension* … *If you give him sugar most of the times he won’t prefer to eat… that sugar will be more important to him than eating food*, *you see*, *yes*.*”* (Interview 7, mother)

Concerns around overweight or obesity as health-related problems were described as things wealthier communities or White people worried about because they were seen as having the means to do something about the problem. Here, the recognition of behavioural mechanisms was more prominent, as can be seen from the quote from one of the fathers regarding going to the gym. What participants described as behaviours or specialised healthcare that might address overweight or obesity were seen as requiring money. Weight was not an urgent health issue to be prioritised if resources were limited, and this was expressed by some as being specific to the working class or Black South Africans.

*“The middle class can actually do something about it in terms of going to gym*, *things like that*. *Paying for that gym*.*”* (Interview 16, father)

*“But with us especially Blacks*, *if a child is big*, *it’s obese*, *it's not a problem as long as they live* … *You know with Whites*, *they take it serious*, *you know they will go to doctors*, *for nutrition and* …*to keep it together*. *Maybe try and get the child to lose some weight but with us*, *we must deal* … *I think they start to taking it seriously maybe when the child start suffering from things like asthma you know*, *he can’t breathe*, *he can’t walk long distance*, *and*, *and*, *and only then*. *When the child is still fine*, *walking around*, *playing around*. *It’s not a problem*.*”* (Interview 15, mother)

Nevertheless, study participants were concerned about the consequences of childhood obesity and expressed that weight could become a health issue if it resulted in diabetes or asthma, hindered children’s mobility or participation, or could not be reversed. Before that, overweight or obesity were not necessarily much of a concern, with a few exceptions among the socioeconomically better off participants who described wanting to make sure their children would not gain too much weight. As mentioned in the previous theme, these parents also raised issues around weight stigma and bullying. This demonstrates that while health may not be the primary or only lens through which weight is viewed, the concerns around stigma and bullying are linked to children’s psychological wellbeing. The possibility of children committing suicide as a result of weight-related bullying was raised, and thus while weight *per se* was not necessarily seen as indicative of health, it could have detrimental consequences to a child’s health and wellbeing.

“*Sometimes you find there are people who…say funny things or hurtful things towards the parents and the kid*. *So if a child understands*, *it's a problem*. *Sometimes some kids commit suicide because of that*. *Like there’s one child in Katlehong who committed suicide because of how they were teasing him at school*. *He was a big child*, *he was very big*.” (Interview 12, mother)

“*It depends on the age*, *I think*. *Firstly*, *when you say your age*, *they’re going to be like*, *‘ah*, *and you’re that big’*. *Like it’s always negative comments…Some people do say it to the child*, *and it’s wrong because it makes them have low self-esteem*. *It really affects them*.” (Interview 13, mother)

## Discussion

The aim of this study was to examine how parents or caregivers of young children in Soweto, South Africa, view children’s weight and size, particularly childhood obesity, and to situate these perspectives in the context of the home environment in which preschool age children in Soweto live. This was done through a reflexive thematic analysis of 16 interviews, which resulted in three themes being developed: growing differently, the ‘right’ way to be, and weight is not health.

Children being small for their age was a somewhat consistent concern across the interviews but many participants also talked comprehensively about potential negative consequences of overweight and obesity. The reasons parents gave for children growing differently reflected the socioeconomic realities of the study setting as the role of genes or other unmodifiable factors were frequently cited as more significant than behaviours in causing variation in weight and size. This is in line with other recent research from Soweto regarding the limited agency people experience when it comes to behaviours or choices that relate to health and weight [32]. Feeling constrained by environmental factors makes it seem futile to focus on behavioural aspects of health or weight, as there may be very little space or capacity for making changes or healthier choices.

This is an interesting contrast to the more individualistic discourses around personal responsibility in obesity prevention in many high-income settings [33], and reflects the broader sense of limited agency that was expressed by participants through comments around class and race in a highly unequal society. These findings underscore the need to consider both equity and effectiveness in developing childhood obesity interventions in low-resource settings, as public health interventions that require a high level of individual agency from intended beneficiaries are unlikely to be effective when individuals are constrained by structural and environmental factors [33].

Children being “fresh” was connected to some positive images like them having everything they want or need. However, the potential health concerns and stigma connected to overweight and obesity meant there was not a consistently positive interpretation of larger body sizes in children. The concerns around negative comments, teasing, and bullying associated with children having overweight or obesity suggest that despite some positive connotations of children’s larger body size, there is also stigma attached to overweight and obesity in this setting. This complexity is important to bear in mind in developing interventions as problematising obesity as a public health concern may exacerbate such stigma. Weight stigma can be very harmful to individuals, as demonstrated both by accounts of study participants concerned about children’s self-esteem and bullying driving children to suicide, and by public health research [34].

Previous South Africa-based research with Black women and adolescent girls has also found views of overweight and obesity to be nuanced, complex and mixed in terms of both positive and negative connotations [35–37]. In contrast, when it came to thinness, participants in the present study did not attribute any positive connotations to it, and parents predominantly described children’s underweight as attracting suspicions about parental neglect. Thinness has tended to carry some stigma in South Africa due to related suspicions of human immunodeficiency virus (HIV) or other illnesses [35]. However, specific fears of HIV stigma did not come up in connection with children’s body size or preferences in any of the interviews in this study, and the stigma around thinness was expressed as being more about what it might say about parenting abilities or a family’s socioeconomic circumstances than health status.

Parents in this urban low-resource setting view children’s weight and size in nuanced and, at times, contradictory ways. Given this complexity and the coexistence of undernutrition and childhood obesity, it may be more relevant to focus preventative efforts on addressing structural factors, and enabling healthy behaviours in a weight-inclusive way, as opposed to targeting obesity alone. All of this would need to be done with a sensitivity towards the risks of weight bias, stigma and disordered eating. Similar discourses are already taking place in high-income settings, and these may also inform health promotion in settings like South Africa [38,39].

Strengths of the present study include the inductive, data driven approach and focus on manifest content in analysing data from research that involved cross-cultural interactions and interpretations. Similarly, collaborating with a fieldworker who understands the study setting from an emic perspective facilitated both data collection and analysis, and guided new questions being flexibly added both after piloting the interview guide and throughout interviews. Cultural differences need to be taken into consideration when interpreting the research findings, despite the interviewer’s experience in cross-cultural settings. Nevertheless, efforts were made to mitigate this limitation by being open about the potential for misunderstandings in the interviews, and frequently checking what exactly participants had meant by what they said. This tended to result in rich and in-depth data in most interviews. Findings and interpretations were also frequently discussed with the fieldworker, and the focus on contextualising participant accounts through field notes enabled partial triangulation of interview findings.

## Conclusions

This study found that parents of preschoolers in Soweto, South Africa view children’s body size and weight through multiple lenses, and their perceptions around how children grow may not centre around health. According to study participants, children should ideally not look too different from peers. While larger body sizes may be favoured to some degree in this setting, there is also a recognition of weight stigma and potential health consequences of severe childhood obesity. There is a need to develop context-appropriate preventative interventions to address the high rates of childhood obesity in South Africa, and this study suggests that rather than addressing weight *per se*, healthy environments and behaviours should be the focus of interventions in order to ultimately address both undernutrition and obesity.

## Supporting information

S1 File Interview guide for in-depth interviews. (DOCX)

S2 File Caregiver questionnaire and observation checklist. (DOCX)
