# Supplementary material for: “The thing is, kids don’t grow the same”: Parent perspectives on preschoolers’ weight and size in Soweto, South Africa
Source: PLoS One. 2020 Apr 6;15(4):e0231094. doi: 10.1371/journal.pone.0231094 (PMC7135213; doi:10.1371/journal.pone.0231094)
Supplement: S1 File — (DOCX) [file pone.0231094.s001.docx]

**S1 Interview guide for in-depth interviews**

**Health behaviours and childhood obesity in context – a qualitative exploration of caregiver perspectives and home settings of preschool age children in Soweto, South Africa**

**Aim**

The aim of this study is to gain a better understanding of how parents or caregivers of young children in Soweto, South Africa, view childhood obesity and related health behaviours, and to situate the phenomenon of childhood obesity, and behaviours associated with obesity, in the context of the home environment in which preschool age children in Soweto live.

**Objectives**

- To explore caregiver perceptions, beliefs, and priorities regarding childhood obesity and health behaviours among preschool age children in Soweto.
- To shed light on the family and home environments in which preschool age children grow up in Soweto in order to contextualise health-related behaviours, and adiposity trends, of young children in an urban South African setting.

**Process**

This is meant to be a very open and exploratory method of interviewing participants. Particularly in the first few interviews, the interviewing technique and focus will develop based on what participants say, and this will inform the approach taken in interviews that follow. Below are broad topics and questions that the interviews are expected to touch upon but it is to be expected that completely new topics (of relevance to the aim) will emerge in interviews, and these can be raised and explored with other participants to find out how pertinent the issues in question are in this setting. The example questions are meant as an illustration of how questions may be presented in the interview but are not meant to be followed strictly if the participant is already speaking freely about topics of relevance. The questions can be formulated on the spot depending on the rapport with the participant but they should be open-ended, and not come across as a test of the participant’s knowledge about health as that may make them uncomfortable, and lead them to answer in a socially desirable way.

The interview visit should start with some polite greetings and small talk, as well as ensuring that the participant is comfortable, has all necessary information, and has given consent. The interview itself will start with the sociodemographic questions, and then move into the more personal and open in-depth interview. It may be good to explain that as a foreign researcher, I may not understand everything and so I am likely to ask for clarification, and that the participant should feel free to explain things assuming I know very little about the topics they are sharing about.

**Topics with example questions**

Children’s weight and size in relation to health

We are interested in your opinions.

- Can you please tell us what you think makes a child of preschool age look healthy? What about unhealthy?
- What is your opinion about a healthy size?
- If you see a very big child, what do you think?
- What about overweight or obesity. How serious or dangerous do you consider it if a preschool child is overweight?

Habits, behaviours, and the neighbourhood

- How easy or difficult is it to get healthy food in your neighbourhood? What do you think about this?
- When it comes to food and meals, can you describe how this works in your household? Who makes decisions, who procures food, who prepares food, and what meals do your child typically eat at home? What about day care, do you provide any food or money for meals at day care?
- If you think about wanting the children in your family and neighbourhood to be healthy, what kind of foods should there be more of, and what kind of foods should there be less of? Why do you think there is too much/too little of ___?
- When your child is at home, what kind of things does he or she do when he or she is awake? Can you show me where in the home?
- What opportunities are there for your child to play outside or be active in some other way? OR Where, other than inside your home, does your child spend time e.g. playing?
- What is this neighbourhood like for children? How do you feel about your child being outdoors?
- Does your child play with toys or equipment? What kind of things? Can you show me?
- Can you describe the sleeping situation in your household? Where do(es) your preschool age child(ren) sleep? When do they go to sleep, and when do others go to sleep? What time does everyone normally wake up?
- This might be obvious from the size of the home.
- “What time do the children sleep?” AND if necessary to ask, also WHERE

Role as parent/caregiver

- I am interested to hear about your experience of being a parent/caregiver. What do you think is the most important thing you do for your child?
- If you think specifically about your child’s growth and weight, what is your role?
- What kind of challenges have you faced as a parent/caregiver when it comes to your child’s health and development?

Aspirations as a parent/caregiver

- What would you like to do differently with your child, if anything?
- What would help your situation as a caregiver/parent in ensuring your child can grow in a healthy way? (careful here to not make it sound like I can provide the help/resources needed)

Examples of probes to be used throughout the interview

What do you think is the reason for that? Why do you think that? What makes you say that? How do you feel about that? Can you tell me more about that? If you could change that, what would be different about the situation? May I check if I have understood you correctly? Who decides that? What makes that possible/difficult/easy/etc.? What is more important out of the things you mentioned, ____ or ___? Why?
