# Supplementary material for: “The thing is, kids don’t grow the same”: Parent perspectives on preschoolers’ weight and size in Soweto, South Africa
Source: PLoS One. 2020 Apr 6;15(4):e0231094. doi: 10.1371/journal.pone.0231094 (PMC7135213; doi:10.1371/journal.pone.0231094)
Supplement: S2 File — (DOCX) [file pone.0231094.s002.docx]

**S2 Caregiver questionnaire and observation checklist**

**Health behaviours and childhood obesity in context – a qualitative exploration of caregiver perspectives and home settings of preschool age children in Soweto, South Africa**

| Today’s date: | Participant #: |
| --- | --- |
| Caregiver’s age: | Neighbourhood (not too specific): |
| Preschool child’s age (or ages): | Preschool child’s gender(s): |

**You and your family**

**(Questions asked by interviewer before interview)**

1. What is your home language? (tick ONE)

|  | English |  | isiXhosa |  | Zulu |  | Tsonga/Shangaan |
| --- | --- | --- | --- | --- | --- | --- | --- |
|  | Afrikaans |  | Sotho |  | Pedi |  | Tswana |
|  | Venda |  | isiNdebele |  | isiswati |  | Other (please state): |

2. Are there other children in your household? How many and how old are they?

| Other children | Child’s  age | Boy or girl? | Relation to you |
| --- | --- | --- | --- |
| 1 |  | 🞆 Boy 🞆 Girl |  |
| 2 |  | 🞆 Boy 🞆 Girl |  |
| 3 |  | 🞆 Boy 🞆 Girl |  |
| 4 |  | 🞆 Boy 🞆 Girl |  |
| 5 |  | 🞆 Boy 🞆 Girl |  |
| 6 |  | 🞆 Boy 🞆 Girl |  |
| 7 |  | 🞆 Boy 🞆 Girl |  |

3. What relationship are you to the preschool child or children in your care? (tick ONE)

|  | Mother |  | Father |  | Grandparent |
| --- | --- | --- | --- | --- | --- |
|  | Aunt |  | Uncle |  | Other (please state): |

4. If mother, ask: Is the father of the preschool child living with you and the child?

|  | Yes |  | No |
| --- | --- | --- | --- |

If father, ask: Are you living together with the mother of the child?

|  | Yes |  | No |
| --- | --- | --- | --- |

5. What is your current marital status? (tick ONE)

|  | Married |  | Living together |  | Divorced |
| --- | --- | --- | --- | --- | --- |
|  | Separated |  | Widowed |  | Never married |

6. What is your highest level of education? (tick ONE)

|  | Grade 6 / Standard 4 and below |  | Grade 7-9 / Standard 5-7 |
| --- | --- | --- | --- |
|  | Grade 10-11 / Standard 8-9 |  | Grade 12 / Standard 10 / Matric |
|  | Tertiary diploma / Certificate |  | University degree |

7. Who in the household works? What do they (/you) do? Do you receive any social grants? Does the other parent pay for maintenance/do they support the children financially?

|  |
| --- |

8. How much time per week does your child spend in day care or being cared for by someone other (who?) than you? (e.g. how many hours per week day, how many hours per weekend day?) Fees per month?

|  |
| --- |

**Toy checklist**

Tick in the boxes next to the toys or equipment you observe in caregiver’s home, and write down the amount/number that you observe (e.g. 2 TVs).

| Soccer/basket/net balls |  | Tennis balls (or small balls) |  |
| --- | --- | --- | --- |
| Basketball/netball ring |  | Skipping rope |  |
| Bean bags |  | Jungle gym |  |
| Bats/racquets/golf clubs |  | Slide |  |
| Sand pit |  | Swings |  |
| Frisbee |  | Tricycle/bicycle/scooter |  |
| Pool or beach toys |  | Trampoline |  |
| Bowls/Skittles/10-Pin Bowls |  | Swimming pool/splash pool |  |
| Other: |  | Other: |  |

| Children’s books |  | Puzzles |  |
| --- | --- | --- | --- |
| Colouring books/pages |  | Crayons/koki pens/pencil crayons |  |
| Lego/Duplo/other building blocks |  | Children’s scissors |  |
| Dolls |  | Fluffy toys |  |
| Board games |  | Dress-up clothes |  |
| Card games |  | Other games: |  |
| Other: |  | Other: |  |

| TV |  | Video/DVD player |  |
| --- | --- | --- | --- |
| Digital tablet, e.g. iPad |  | Smart phone |  |
| Video games, e.g. Playstation, X-Box |  | Satellite dish (for TV) |  |
| Computer |  | Internet access |  |
| Other: |  | Other: |  |

**Overall observations**

After the interview, the interviewer should write detailed field notes about the interview, interactions with the participant, any observations about the child and participant and their interactions, the home environment, the neighbourhood, and anything else that comes to mind that could potentially be of any relevance for contextualising the interview. These field notes can inform a probing strategy and topics to follow up on in other interviews.
